# Perceptions of Health Communication, Water Treatment and Sanitation in Artibonite Department, Haiti, March-April 2012

**DOI:** 10.1371/journal.pone.0142778

**Published:** 2015-11-12

**Authors:** Holly Ann Williams, Joanna Gaines, Molly Patrick, David Berendes, David Fitter, Thomas Handzel

**Affiliations:** 1 Emergency Response and Recovery Branch, Division of Global Health Protection, Center for Global Health, Centers for Disease Control and Prevention, Atlanta, Georgia, United States of America; 2 Travelers Health Branch, Division of Global Migration and Quarantine, National Center for Emerging and Zoonotic Infectious Diseases, Centers for Disease Control and Prevention, Atlanta, Georgia, United States of America; The Australian National University, AUSTRALIA

## Abstract

The international response to Haiti’s ongoing cholera outbreak has been multifaceted, including health education efforts by community health workers and the distribution of free water treatment products. Artibonite Department was the first region affected by the outbreak. Numerous organizations have been involved in cholera response efforts in Haiti with many focusing on efforts to improve water, sanitation, and hygiene (WASH). Multiple types of water treatment products have been distributed, creating the potential for confusion over correct dosage and water treatment methods. We utilized qualitative methods in Artibonite to determine the population’s response to WASH messages, use and acceptability of water treatment products, and water treatment and sanitation knowledge, attitudes and practices at the household level. We conducted eighteen focus group discussions (FGDs): 17 FGDs were held with community members (nine among females, eight among males); one FGD was held with community health workers. Health messages related to WASH were well-retained, with reported improvements in hand-washing. Community health workers were identified as valued sources of health information. Most participants noted a paucity of water-treatment products. Sanitation, specifically the construction of latrines, was the most commonly identified need. Lack of funds was the primary reason given for not constructing a latrine. The construction and maintenance of potable water and sanitation services is needed to ensure a sustainable change.

## Introduction

In October 2010, Haiti’s Ministère de la Santé Publique et de la Population (MSPP/Haitian Ministry of Public Health) saw a rise in cases of acute watery diarrhea in Artibonite and Centre Departments (locally defined administrative regions). These initial cases were confirmed as *Vibrio cholerae* O1, serotype Ogawa, biotype El Tor, and marked the beginning of the current cholera epidemic. As of January 7, 2014, this epidemic had resulted in 745,588 cases and 8,972 deaths nationwide [1]. The spread of cholera was exacerbated by risk factors such as poverty, immunological naiveté, poor water and sanitation infrastructure, high population density, and population dislocation [2–4].

To reduce transmission of cholera and other diarrheal illnesses, access to safe drinking water is essential [5]. In response to the outbreak, MSPP, the Direction Nationale de l’Eau Potable et de l’Assainissement (DINEPA/Haitian National Water and Sanitation Directorate), and other agencies focused efforts on safe water supplies and hygiene promotion. This included mass distribution of water treatment supplies for the disinfection of drinking water at the household level. Many Haitians live in isolated rural areas and lack access to piped water systems, and household water treatment (HWT) was a potential measure for improving access to safe water.

A cornerstone of the emergency response to cholera in Haiti has been the free distributions of household water treatment products to encourage HWT[6]. Household use of water treatment products was high among those that received them; in one survey the use of water purification tablets increased from 29% before the outbreak to 87% one month after [7]. There were multiple distributions of water treatment products since the outbreak. Families in a recent survey in the Northwest Department reported using multiple household water treatment products from prior purchases or distributions: over one-third of families had three or more products in the home at the time of interview and over 10 different products had reportedly been used in the Northwest department in the year since the outbreak began [8]. As a result of these distribution campaigns, public awareness of the importance of safe water and demand for water treatment products is expected to be higher than before the outbreak.

However, as the number of cholera cases has decreased, the strategy has shifted from emergency distributions of free water treatment supplies to long term and sustainable access to safe water. Although water treatment products have been widely distributed in Haiti, the specific knowledge, attitudes and practices (KAP) related to them have not yet been examined. Additionally, behaviors and motivations influencing regular use of particular household water treatment products have not been assessed; this information is necessary to understand long term adherence to treatment products and practices. This information can improve future interventions with regards to their acceptability and sustainability, improving adherence and ultimately health outcomes. Building upon an earlier Centers for Disease Control and Prevention(CDC)-supported KAP survey conducted during November 2010 [7], qualitative operational research (a series of focus group discussions [FGDs]) was undertaken in similar areas in the Artibonite Department during the ongoing outbreak to better understand what type of health communication had occurred in this area, the best methods to reach affected populations, people’s perceptions of water treatment and what constituted ‘safe’ water, as well as perceptions relating to defecation and use of latrines. This qualitative study was in conjunction with a quantitative baseline assessment of household water treatment products and water use in the same geographical areas [9]. This report concentrates on the findings from the qualitative focus group discussions.

### Objectives

The main objectives of the qualitative study were:

1. To determine what water, sanitation, and hygiene messages received by households in Artibonite were being retained and used.
2. To describe the current use and acceptability of household water treatment products in communities in Artibonite.
3. To describe types of drinking water sources and access to sanitation facilities in Artibonite.
4. To describe knowledge, motivation, barriers and behaviors surrounding water treatment and sanitation at the household level.
5. To provide a baseline to monitor future efforts in expanding access to safe water in Artibonite over the next 2–3 years.

## Methods

An interview guide was developed for focus group discussions (FGDs) from a review of pertinent literature and anecdotal data from previous CDC cholera-related field work[7,9]. The guide was translated into French and Haitian Creole, with special emphasis on ensuring that the Haitian Creole reflected rural phrases. The guide was then back-translated from the respective languages into English and modified as necessary. The guide contained questions about how health messages are best received, whether people treat their water (and, if not, why not), and what treatment products are used and/or preferred in the communities. The guide also included questions pertaining to sanitation, including the number of individuals that owned a latrine, reasons and perceptions surrounding latrine ownership and usage, and what assistance was needed for individuals to build latrines.

### Ethics Statement

The FGD guide and protocol were reviewed by the CDC Institutional Review Board and determined to not qualify as human subject research. Prior to each FGD, the facilitator of each team obtained verbal informed consent for participation in the FGD and permission to manually record anonymous responses. Verbal consent was obtained as the majority of the participants were illiterate. The consent was noted and dated on the first written page of the interview guide by the facilitator. The informed consent procedure was reviewed by the CDC IRB and approved during their determination of 'non-research' status.

### Recruitment and Training of Staff

Ten potential field staff members were pre-identified from a partner non-governmental agency, with the requirement that all of them had the ability to speak and write both French and Haitian Creole. A two-day training was held in Gonaives to train the field staff in qualitative methods, sensitize them to the larger intent of the project, and to allow them time to practice interviewing and recording of data. Training materials, developed by the qualitative co-investigators, were translated from English to French. The co-investigators observed the interviewing and recording skills of the field staff and, based on those observations, selected the two FGD facilitators and four recorders. In addition, two staff members that were fluent in Haitian Creole and proficient in English were chosen to be translators for the co-Investigators and team leads. Facilitators, recorders and translators were matched to FGDs by gender. They translated for the co-investigators as needed during the FGDs.

### Pilot Testing and Procedures

Following the training, the FGD guide was pilot tested in a peri-urban area outside of the town of Gonaives, with one female and one male FGD. At the completion of these groups, the field team discussed each of the questions to determine if the question was understood and to identify any final modifications.

Focus group discussions comprised of adult men and women were conducted in rural areas of the Artibonite Department, with one additional FGD in the town of Gonaives, aimed at understanding the perceptions of community health workers (CHWs). All group interviews were conducted in Haitian Creole. Several participatory exercises were done during the discussions. Participants were shown 14 laminated card pictures of different methods used to treat water, including water treatment tablets, filters and chlorine products, and asked to rank the top three in order of preference and to explain why they were selected as the top products. Participants were also asked to draw a map using a large piece of paper and colored markers of places where members of the community defecated, illustrating key features of their villages (such as a market, church or river) and marking the areas where there were feces (e.g., where they had defecated or observed feces or other people openly defecating).

Each FGD team consisted of one facilitator, two recorders who wrote down responses, one translator, and one co-investigator from CDC. At the end of each FGD, debriefings were held with field staff members and the two co-investigators to assess any concerns or problems identified in the field. Responses to the questions were recorded in Haitian Creole and then orally translated during the team debriefings. Team debriefs were also used to note trends in the data and to gain consensus on language translations.

As a result of these daily debriefings, on the second day of conducting FGDs, it was decided that a question regarding water treatment products that were not included in the ranking exercise would be dropped as the question was not understood by the participants. Debriefings continued throughout data collection to monitor data quality, but there were no further modifications to the discussion guide.

Field sites were chosen from the same cluster areas that were identified for the associated quantitative baseline household survey [9]. Nine locations were chosen randomly from the pre-identified clusters, with a few clusters not included in the selection due to logistical difficulties in reaching those areas. Ten FGDs were held in the northern areas of Artibonite and the remaining eight were conducted in the southern areas. In each of these locations, a male and a female FGD was conducted, with the exception of one location that did not have a male FGD, for a total of 17 community FGDs. During that time, a mixed-gender FGD with CHWs was held concurrently in the city of Gonaives. At the conclusion of each FGD, participants were given a snack and encouraged to talk freely about any other issues or to voice questions.

In advance of the training and field work, CDC staff working on the associated household survey met with partner agencies to select the field sites and to provide guidance for the FGD participant selection. As brigadiers (community-based health workers specifically assigned to work on cholera) knew the selected sample sites, they were chosen to select the FGD participants prior to the start of the evaluation. Brigadiers were asked to select six to twelve individuals who resided in different areas of their respective catchment areas, did not know each other and were between 18–40 years of age. Once selected, the participants were given instructions about the date, time, and location of the FGD.

### Analysis

Local translators, pre-selected by a partner NGO, were used to translate the hand-written data from Haitian Creole to English. If discrepancies were noted in their translation, a third translator examined the transcript for clarity. CDC co-investigators also examined the original Creole to check for discrepancies in what was written across recorders.

Data were analyzed using frequency counts and conventional content analysis [10]. The two CDC co-investigators coded the data and examined it for themes. Frequency counts of responses (when appropriate) were used to describe the strength of those responses, within and across groups. Disagreements between coders were rare: if there was a discrepancy between the two coders, the question was reviewed and discussed until consistency was obtained. Data were coded by site to allow analysis by location (urban vs. rural), and by gender. Direct quotations written during the FGDs were used to illustrate points of view of the participants.

## Results

A total of 18 FGDs were conducted between March 30^th^ and April 4^th^, 2012 in Artibonite Department. Fourteen FGDs were held in remote, rural areas (up to two hour drive from a city), two were in a semi-rural area (within 30 minutes of a small city), one in a peri-urban setting approximately 15 minutes from a city and the CHW FGD was held at a partner NGO headquarters in Gonaives. Of the FGDs, there were nine female groups, eight male groups, and one community health worker group (mixed gender). Most groups had 10 participants and, while specific demographics were not recorded, varied across ages. In spite of the participants’ age differences, most groups were very vocal across participants and all members of the groups participated in the ranking and mapping exercises. In some groups, participants directed the field team to draw the maps with input from the group (likely due to low literacy).

### Health Education and Communication

All participants indicated that they knew their CHWs or brigadiers; the vast majority of participants (all male groups and 7/9 female groups) described meetings with the CHWs/brigadiers within the previous five months. Water treatment and sanitation (including use of latrines and messaging related to cholera) were discussed in the majority of groups during these meetings.

When asked the best way to reach communities with messages in general, the female FGDs identified the use of health workers, including CHWs, brigadiers, and health and sanitation agents. For the male groups, the majority noted that using a megaphone was the most effective means of communication, followed by equal mentions of church, SMS and radio. However, when asked about the best ways to communicate health messages specifically, there were no gender differences. Both male and female FGDs identified use of megaphone and health workers going door to door as the best ways to reach the communities.

Both male and female groups described hearing similar health messages; namely, messages related to health promotion. This included messages such as: keeping yourself and your children clean, using hygienic practices, treating children promptly when sick, washing hands, treating water, and food handling (washing fruits and vegetables before eating or the need to cook food properly).

As described in a female focus group discussion: “*We have to wash your hands before you eat*, *put treatment in your water*, *and keep yourself clean*.*”*

All participants said that they understood the messages and the vast majority (9/9 female groups and 7/8 male groups) indicated that the messages were not confusing and perceived to be beneficial to the communities. All male groups and 8/9 female groups noted that they changed behaviors after hearing the health messages, with hand washing as the most commonly reported changed behaviors. The rural group that indicated that they did not change behaviors noted that they were unable to modify behaviors due to logistical constraints: they did not have easy access to water and had no latrines in the area. Various times were noted to be important when washing hands–after toileting, upon returning home from the fields or the markets, and before eating or cooking.

As one group noted: *“Health workers have little resources to make changes as they pay them too little but*, *with what they have*, *they make some changes*.*”*

#### CHWs Perspectives

The CHW FGD noted that they had monthly meetings with their communities within the past three to six months, averaging about ten people per meeting. Key areas discussed included: a) need for water treatment, b) prevention of cholera, and c) nutrition, including malnutrition and safe food handling practices. Nutrition and cholera-related information included how to make oral rehydration solution, treating water, and how to feed infants. They also identified going door-to-door and using a megaphone as effective ways to send messages, as well as using flyers or posted signs and attending church meetings. According to the CHWs, the most effective means of communication was going door-to-door.

In terms of cholera, CHWs reported observing changed behaviors in their communities since the outbreak began. Notably, they perceived that community members were drinking more treated water, there were more latrines in their areas, rates of diarrhea had decreased and safe food handling had improved, including washing of fruits and vegetables before eating them. They also noted improved communication and collaboration between the CHWs and ‘bokors’ (traditional healers).

### Water Treatment

Communities used two methods to ascertain whether water was safe for drinking: either to use a source of water that was known to be treated (such as piped or kiosk water) or to have the water deemed safe by an authority. Participants described health workers coming to test water sources and then posting signs to inform the communities whether they needed to treat the water or not. All groups noted that they learned of these messages from health workers. When asked was there a best method to tell if water was safe, all groups said ‘yes’ and identified treated water (particularly by use of Aquatabs [water chlorination tables]) as the safest water to drink. Boiled or filtered water, kiosk water, water treated by a chlorinated product (other than Aquatabs), spring water (verified to mean water from a natural spring) or piped water, were all perceived to be safe. A few participants also described additional methods of water treatment, such as solar disinfection and adding lemon to untreated water.

Although the female groups agreed that people in their respective areas routinely treated water, the male groups did not reach consensus on this issue. However, both female and male groups were quite consistent in identifying use of Aquatabs and then some form of chlorine (Clorox, “Jif” or loose granulates of chlorine) as the methods most commonly used to treat water.

When asked why people did not treat their water, women stated that it was due to either being lazy or careless or simply not believing in the threat posed by unsafe water. For men, they identified the same theme of disbelief and also a lack of water treatment products. Comments similar to the following were heard throughout several groups as participants explained the disbelief:

“The microbe that can kill Haitians is not yet existing.”

“My grandparents drank the water and they lived. I have been drinking it untreated since birth and haven’t gotten sick.”

Both men and women identified various ways to obtain water treatment products, with more women than men identifying buying them at the market as an option. Other sources noted by both groups included donations from non-governmental organizations (NGO), health workers or community leaders (‘casaks’). People learned about sources of water treatment products from their health workers or through donations.

Regarding water treatment products, a women’s group explained: *“The agent of health explained that [the need to treat water]*, *they made training for us*, *when the cholera was [here]*, *they gave it to us*, *now we buy it*.*”*

The vast majority of all the groups (8/9 female and 8/8 male groups) agreed that water treatment products were difficult to find now (as opposed to the “time of cholera”). Throughout various groups, time was divided into “time of cholera” and “now.” Cholera was perceived to be mostly gone, although in participants, when asked in more detail, noted that as soon as the rains came again they expected to see a return of cholera. “Time of cholera” referred to several months prior to our FGDs when cases of cholera remained high. There was uniform agreement that there were entire periods of time when products were not available due to the cessation of donations (given during the “time of cholera”).

A female participant noted, *“The people who used to give it [water treatment products] do not come*, *and the agents of health do not have any more in this moment*.*”*

A male participant in a different group noted the same issue: “*If it’s not the health agent*, *it is not easy to find*, *it is not available*.*”*

If no specific drinking water treatment products were available, women reported boiling water, followed by use of non-specific chlorine products (liquid or loose granule form). For men, slightly more than half of groups said that they would drink the water as is, followed by boiling water.

Women and men identified the same products as being the ‘best’ water treatment products–Aquatab was most commonly mentioned, followed by Jif (liquid chlorine). These products were described by participants as being the easiest to find and use.

Almost all groups were consistent in identifying that the pharmacy was the most trusted vendor for water treatment products, with 8/9 female groups stating that products at the pharmacy were not expired (in contrast to this, the women noted that market vendors would be willing to sell expired products). Male groups were split in their reason for why they trusted pharmacies. They agreed that pharmacies would not sell expired products but noted, as well, that the pharmacy staff was trained. One male group commented that pharmacies do not have any incentive to sell expired products, while a women’s group said that, because they did not sell expired products, it was safer for illiterate people to purchase items in pharmacies, as they would not be deceived.

When asked how many participants bought kiosk water, 25 women (28.7%) and 28 men (35.9%) reported purchasing kiosk water. Both women and men had the same reasons for purchasing kiosk water–it was pre-treated (and did not require any further treatment efforts) and was perceived as being protective for their health. All groups consistently agreed that, for people who work in gardens or fields away from their homes, the vast majority carried bottles of treated water from home or carried Aquatabs to treat water as needed. More men than women suggested that water that they carried from homes was not treated.

#### CHWs Perspectives

The CHWs were asked slightly different questions about water treatment in order to gain a broader perspective on behaviors associated with treating water.

Community health workers described two methods that community members used to ensure safe water: drinking water from covered wells or drinking spring water. Some CHWs reported that their spring water was treated, while others did not. They also discussed the perception held by some community members that ‘good will’ would protect them; thus, they did not have to treat their water. There was also no consensus among the group as to whether communities used the same water source for all activities of daily living. The primary source for water in their respective communities was mainly spring water, as that was all that was available. They also noted that people often had to search for local water sources. There was no consensus about whether the water used by people who worked in gardens/fields was treated or not–some felt that individuals carried bottles of treated water, while others said that they drank from any source.

When asked what methods were used by communities to treat their water, the most common methods included Aquatab, liquid or powdered chlorine and filters. CHWs stated that Aquatab was used more often than the other methods because most people understood the Aquatab directions. Community members most often got these products through donations or purchasing them. CHWs noted that during health education the importance of buying water treatment products from a pharmacy was stressed. There was no consensus on what communities did when donated products were not available–some purchased water treatment products, while others drank untreated water.

There was also no consensus among CHWs regarding the best distribution system for water treatment products. Door-to-door visits were mentioned but not everyone agreed that this was the best method. One participant mentioned the need for each locality to have its own water treatment system, while others suggested working with small groups and stressing prevention messages before any distribution. Another question asked the best way to encourage people to purchase water treatment products. The CHWs responded that by stocking the products in local stores and using CHWs to demonstrate the products would encourage sales but they also noted that NGO distribution (free distributions) was a good distribution system.

Similar to the results from the female and male FGDs, CHWs noted that the preferred treatment product was Aquatabs in almost all localities and that people treated their water ‘always’ after a donation or after hearing water treatment messages. They also agreed that people did not treat water due to negligence/laziness, lack of product or disbelief in the idea that water needed to be treated.

### Sanitation

The last area of inquiry dealt with perceptions pertaining to sanitation. All participants could describe health messages that they heard in terms of sanitation within the last year. Both women and men most commonly described hearing sanitation messages pertaining to the need for hand washing. Women also mentioned cholera prevention methods, avoidance of open defecation, and safe food handling (also mentioned by men). The vast majority of women’s groups (8/9) identified ‘microbes’ as the cause of diarrhea in children, adolescents and adults, while men noted unsafe water and teething as the cause of diarrhea in children and unsafe water and inappropriate food handling as causes of diarrhea for adolescents and adults.

When asked whether latrines were commonly found in their respective communities, answers were mixed, although slightly more groups (9/17 FGDs) said no (4/9 FGDs for women, 5/8 FGDs or men). Only one female group in the northern part of Artibonite (Ennery site) had 10/10 women saying that they owned a latrine. Just under half of female participants (46%, 40/87) reported owning a latrine, with five women stating that it was not a ‘real’ latrine as it was only an uncovered hole in the ground. Slightly more men (57.7%, 45/78) stated that they owned a latrine but, like the women, 26 noted that it was only an uncovered hole in the ground. Discounting those who described their ‘latrines’ as ‘open uncovered holes,’ the percentage of latrine ownership drops to 40.2% for women and 23.4% for men.

Most groups agreed that latrines were private and not shared, but it was not clear if this question was understood by participants. While participants described latrines as ‘private’ for each family, they noted that people passing by (a guest) would also use it. All participants were in unanimous agreement that the reason why there were so few latrines was simply lack of money. Female participants noted the latrine owners were perceived to be wealthier or to have been given more opportunities (such as having a NGO build a latrine for them), while male participants noted that there was some jealousy directed at latrine owners as they were perceived to have more money. In spite of recognizing latrine owners as having more wealth, the majority of the male FGDs noted that latrine ownership did not confer more status or prestige in the community. In contrast, the female FGDs could not come to a consensus on this question of status/prestige.

There was a marked gender difference with identifying who made the decision within a family whether to build a latrine or not. Eight out of nine female FGDs said it was a shared decision between husband and wife, while 8/8 male FGDs said the husband made the decision

When asked what could be done to get more people to build their own latrines, the most common response across groups was economic aid/more help. The need for social mobilization around this issue was mentioned briefly and one group said that ‘cash for work’ programs were needed. All groups stressed the need for outside intervention–through donations or unspecified ‘help’–as the primary way for latrines to be built.

In response to a question about health risks if people did not use latrines, all the female groups noted that people would get sick, with cholera and malaria mentioned most often of the specific illnesses identified. For men, cholera and typhoid were the most frequently identified consequences of not using latrines.

Participants were asked to identify where people defecated if they did not have access to a latrine and, universally, the answer was ‘everywhere.’ Examples were given such as: a) by the roads, b) on the rocks by the river, c) in the river, d) by churches or schools, e) on the ground, etc. A few groups described buying a black plastic bag for two gourdes in which they could defecate at night without having to go outside [later explanations included the fear of encountering ghosts if one went outside the house at night].

Each group was also asked to draw a community defecation map and the maps mirrored what the groups verbally described–open defecation throughout the community, including other areas such as in cemeteries and by cock-fighting rings. Participants exhibited no shyness in talking about open defecation and comments included:

“If it is time to poo, you poo anywhere as you cannot keep it.”

“When we walk on the mountains, we walk on the toilets.”

#### CHWs Perspectives

In terms of latrines, CHWs related that there were few latrines in their respective areas. Similar to what was voiced in the other FGDs, they noted that latrines were often saved for use by local leaders or guests. The key factor that limited latrine ownership was lack of money, although they also mentioned that NGOs had built some latrines for individuals who could not afford to pay for a latrine independently. Perceptions of those who owned latrines were that they were wealthier than others or more fortunate in that certain families received a latrine from NGOs. Sanitation was perceived as problematic, with open defecation occurring everywhere in their communities. They noted that health care clinics with which they were associated did have latrines but they did not comment further on the status of those latrines. When asked how they would encourage people in their respective communities to use latrines, the question was not answered directly other than to say that people had to be taught how to use latrines.

### Community Priorities

As a way to ensure completeness of information, participants were asked to describe the top three priorities for areas of improvement in their communities. Nearly all of the community focus groups (15/17) identified ‘sanitation’, specifically latrines, as a top priority for their community. The female FGDs mentioned latrines and treated water as the top priorities, followed by access to a health center. Men listed treated water, followed by access to a health center. Men also identified wanting agricultural assistance, including irrigation, reforestation, and agricultural credit.

### CHWs Perspectives

Priority needs identified by CHWs included clean water, latrines, health centers and schools.

#### Post-FGD Informal Discussions

Following the formal end of the FGDs, during the snack period, participants were encouraged to offer any additional information that the team may have missed or to ask questions. One female FGD (approximately 15 minutes outside of Gonaives) complained that many people and different groups have been out to talk with them about community concerns but that there was no visible follow-up to correct the concerns. The CHWs also discussed the need for follow-up, particularly related to water treatment, sanitation and development of new schools, as they noted that many of them were working in very rural, isolated areas with little access to basic amenities, such as clean water and sanitation.

## Discussion of Results

There was marked consistency in results from women and men related to health education and communication, treatment of water and the reported need for external economic aid in order to build latrines. Please see Box 1 for summary of key findings.

**Box 1. Summary of Key Findings**

- Health messaging in relation to WASH was retained by the community, especially related to the need for hand washing, use of latrines and proper food handling.
  ○ An increase in hand washing was the most commonly reported behavior change as a result of messaging.
- CHWs were perceived as the most valuable source of health information.
- Communities ascertained the safety of water sources by using a known treated source or waiting for authorities to verify that a source was safe.
- The commercial product ‘Aquatabs’ and other forms of chlorine were the most commonly reported products used to treat water.
- Participants noted that there was a paucity of water treatments products in their area.
- Pharmacies were perceived to be most trusted vendors for water treatment products.
- Lack of latrines and open defecation were commonly reported.
- Lack of funds was the primary reason cited for an inability to build a latrine.

Health messaging and education were well received by the communities at large, particularly in regard to understanding the need for hand washing, use of latrines and proper food handling. Community health workers were reported to be active, recognized by their communities, and credited for providing correct information about general health care, water and sanitation issues, proper food handling and prevention of cholera. There was remarkable consistency by gender and across geographical locations in health messages that were received and understood, which resulted in a reported changing of behaviors (most notably handwashing). The health education directly addressed the public health issues regarding the cholera epidemic and mirrored results from a KAP survey done in December 2010 in Port-au-Prince and a household survey completed in 2012 in Artibonite [11]. The consistency of the messages received and the reported clarity of the messages may reflect the intensive and standardized training of CHWs throughout Haiti during March 2011, which resulted in at least 1,170 CHWs receiving cholera-specific training [12].

Although some minor differences existed in terms of the best ways to deliver health information to communities, men, women and CHWs all agreed that having health care workers going door to door or using a megaphone was the preferred means of reaching the public with health messages. In contrast, recent results from a KAP survey indicated that urban dwellers in Port-au-Prince preferred television and trucks with megaphones as the best ways to communicate health messages [11]. This discrepancy may reflect the poverty of the areas we sampled, where televisions were few and far between.

Reliance on health care workers (broadly defined here to include community health workers, people working in pharmacies, sanitation and health agents and those working in health care facilities) extended beyond health education to that of perceptions of water quality. Community health care workers were trusted to verify water quality for communities and to provide education about the need for clean water and improved sanitation. This is consistent with findings from a recent study in Malawi [13] where ongoing interpersonal contact with trusted health care and community health care workers through health education and home visits enhanced the use of home water treatment products.

Findings indicated that the need for water treatment was well understood and that these rural Haitians could identify an array of treatment options. Universally, Aquatab and liquid chlorine were identified as the preferred methods to use due to availability and ease of use. Our findings were consistent with a recent quantitative survey conducted in the same area [9]. Less than half of the participants bought kiosk water, even when water treatment products were not available. Even fewer participants (3.4%) in the abovementioned survey reported buying kiosk water [9]. Participants described fluidity with switching among treatment options, including home-based remedies like solar radiation or use of lemon. Other studies have found that, even with trust in and positive perceptions of specific water treatment products, consumers still switched back and forth between products [13].

Participants were adamant in their descriptions of the paucity of water treatment products currently available and noted that they had been used to receiving donations of the products through the government or NGOs. Even though some were willing to buy products in the local markets, participants maintained that treatment products were simply not available. The associated household survey for this project found similar results [9]. Given the high level of trust in the pharmacy system, this could be an ideal avenue to combine product distribution with continued health messaging and education on why water treatment products are important, even during periods when cholera is not prominent.

Without the presence of water treatment products, it is unclear whether or not the willingness to treat water will remain, particularly if the number of cholera cases remains low. In general, factors that influence the acceptance and adaptation of home water treatment products or why household water treatment is not widely practiced in vulnerable, low-income settings are not well understood [14,15].

A critical review of behavior change research on point-of-use water treatment interventions noted that only 27% of the 26 studies showed maintenance of longer term adaptation of point-of-use water among at least half of the population, with a median duration of 14.5 months at the end of follow-up [15]. This low usage occurred in spite of products being available. In a trial in Bangladesh where poor households were given free trials of multiple water treatment products, no product was used by even 30% of the target population, in spite of educational efforts that were combined with the distribution [16]. The authors urged that behavioral research in this area has to extend beyond looking at cost, product information/education and variation among products in order to explain choices made at the household level. A 2010 review by Figueroa and Kincaid [17] found that water treatment is often inadequately conducted or treated water is improperly stored resulting in contamination; individuals who try water treatment inconsistently or inadequately may then fail to gain any real or perceived health benefits. Decline in use of products over time may be due to inability to purchase products, failure of the device or choice [18]. With the current study, few reasons were given for why water was not treated, other than products not being freely available. In Haiti, longitudinal research that combines water quality testing and qualitative interviewing about behaviors (specifically, motivation and choice) is needed to better inform programmatic decisions about how to best target at-risk populations, select an appropriate distribution mechanism and sustain home water treatment practices.

Various authors [15,18,19] have noted that, due to various methodology problems, the published literature offers little guidance for implementing point-of-use water treatment interventions. Suggestions for future research in this area included the need to understand patterns of use over time, conducting formative research to explain contextual factors that influence long-term adaption of water treatment products (including but not limited to factors such as: preferences, choices, household-level decision making influencers, and aspirations of the vulnerable populations), and incorporating behavioral theories (such as the Health Belief Model [20]) in the development of the project design.

Perceptions of the threat of cholera were strongly tied to seasonality, with an assumption that cholera would return when the rains returned. Seasonality may also play a role in terms of perceived risk regarding water source safety in relation to cholera [9]. In Malawi, seasonality (e.g., perceived as the need to treat water only during the rainy season) was also cited as a factor for intermittent use of water treatment products [13]. This has important implications given that the cholera outbreak in Haiti continues to claim lives, regardless of the season, and the need for an accessible safe water source is constant.

In contrast to the majority of participants that understood that illness was associated with unclean water, a small number of individuals did not perceive unclean water as a threat to health, in spite of having large numbers of cholera cases in their Department during the epidemic. This is consistent with other research suggesting that diarrhea is perceived to be an outcome affected by multiple factors, not all of which are related to hygiene [17]. Other risk factors such as subjective norms, perceived vulnerability, perceived severity and factual knowledge all play a role in changing behaviors [17,19]. In our investigation, some of the participants clearly did not perceive a risk to their health from unclean water. They lived in areas where cholera numbers were high and health education regarding cholera prevention and risks related to untreated water was perceived as abundant and clear. Despite these exposures, disbelief remained. Additional behavioral research would be needed to better understand the factors underlying their perceived minimal vulnerability in the face of a large epidemic.

Increased access to improved sanitation and having more latrines in the community were perceived as having positive impacts on communities. Jenkins and Sugden [21] noted that cholera epidemics may bring short-term changes in household behaviors but reasons to adapt improved sanitation often are not associated with public health concerns at the household level. Rather, factors such as privacy, improved property value, improved comfort and safety and dignity and social status may be more pivotal in the decision to build latrines. Given the level of poverty in Haiti, strategies would need to be modified to that particular context after adequate baseline data have been collected to determine if, when and under what circumstances the poorest segment of the population chooses to build latrines. To meet the needs of the poor, latrines would need to be affordable and, perhaps, supplemented through community-based savings and loans programs or other economic assistance programs.

It was not clear from our results why more women than men in the same residential area identified themselves as latrine owners. There was a marked discrepancy between women and men regarding who decides whether or not to build a latrine. It has been suggested that sanitation may be more important to women than men; however men, often have decision-making authority and may hold different priorities [22].

In spite of the high recognition of the need for more latrines, the building of latrines was not seen as a shared community problem. Few focus group discussions, including that of the CHWs, offered any community-based strategies to begin to tackle this problem. Extreme poverty, devastating effects to the environment and infrastructure from the 2008 flooding and 2010 massive earthquake, coupled with large donations of water treatment products in order to curb the spread of cholera, may have contributed to a culture of dependency in which individuals believe that others are responsible for fixing their own social situations [23]. A culture of dependency is an extremely complex situation that often reflects historical repression, unstable political situations, corruption and other social factors that cut across sectors. Public health interventions will need to allow time for communities and individuals to shift gradually from dependency to enhanced individual determination. In order to fully meet the needs of the Haitian people, public health interventions need to be conceived of and developed using models that support participatory decision making and governance [24].

In a study that examined factors that influenced the decision to install pit latrines in rural areas in Benin [25], latrine adaptation was not influenced by health considerations. Rather, a sense of prestige and convenience and comfort were the stronger motivators. These reasons differed from our findings, in which there was no strong association between latrine ownership and perceived status, only a common recognition that those who owned latrines had more money. Health consequences to not owning latrines were recognized but our questions failed to elicit any other motivational factor affecting latrine ownership, other than poverty. Additional research would be needed to have a greater understanding of the segment of the rural population that might be early adaptors of latrines, should resources be available.

As noted earlier, this study complemented a quantitative household survey that described the types of quality of water sources and knowledge, access and use of household water treatment products [9]. Triangulating data from quantitative and qualitative research strengthened the research process as it increased the validity of the data. While the quantitative data offered numerical measures, the qualitative data provided a broader contextual understanding of the factors that influenced the quantitative findings. Qualitative methods allowed for more in-depth discussion of pertinent topics. For example, the quantitative findings identified primary sources of drinking water. The FGDs added information on how participants ascertained whether sources of water were potable, described home methods of treating water, and identified beliefs that influenced perceptions about water quality. Please see Box 1 for a summary of the key findings of the study.

## Limitations

Haitian Creole is primarily a spoken language that is written phonetically and not well standardized, which makes translation difficult as spelling of words is inconsistent. Translation was the biggest limitation, which hindered our ability to understand fine nuances. There is a severe shortage of individuals trained in qualitative research methods, particularly those with experience in interviewing and the use of probes to gather additional information. Although the co-Investigators reinforced the concepts from the qualitative training on a daily basis during the debriefings, facilitators had limited ability to use probes or ask additional questions to elicit more comprehensive information from the FGDs. Review of responses to several questions indicated that the questions were not well understood by participants and, thus, those questions were dropped from the analysis. In retrospect, additional questions would have been useful for a broader understanding of hygienic practices, such as the use and availability of soap or the availability of firewood or alternate fuel sources that are needed in order to boil water.

There may have been biases related to participant selection. For example, participants may have been motivated by an expectation of services. It is important to remember that the FGD participants had been selected by the brigadiers. This may have influenced individuals reporting that they knew their CHW/brigadier as well as over reported the role of CHWs/brigadiers in delivering health messages.

Self-reporting occurred during the FGDs. When asking about behaviors, there is an inherent social desirability bias, in which participants answer in a manner that they hope will be perceived as good or positive by those asking the questions. This bias may be mitigated somewhat by comparing the qualitative results with the associated quantitative results of the household survey, which includes objective data, such as total chlorine levels that indicate if water was ever chlorinated. Self-reports of use of water treatment products can be examined against the total chlorine levels.

In terms of sanitation questions specifically, there were also a few limitations. The study did not offer a standardized definition of latrines and it was unclear how ‘latrine’ was translated. As well, there were no observations of latrines made during our time in the villages. The questions did not elicit any information on other factors that may impact the decision to build a latrine, such as soil quality or technical complexity.

## Recommendations and Conclusions

In spite of efforts made post-2010 Haiti earthquake and the recent cholera epidemic, many Haitians, particularly in rural areas, remain without access to clean water or improved sanitation. All of our participants cited poverty as the primary barrier to building latrines.

Additional operational research is needed in Haiti to determine if and what additional factors, other than cost, play a role in choosing to build latrines. Once those factors are identified, then appropriate sanitation education and marketing can be developed.

Findings from this study indicated rural women and men in different physical locations within Artibonite Department held very similar perceptions regarding health education and the impact on behaviors, water treatment products and availability, and sanitation. Community health workers were perceived in a very positive manner as professionals who were trusted to offer clear and understandable health messages, serve as authorities for verifying water quality, and provide water treatment products that were not expired. Our data are consistent with other recommendations [26] to use community health care workers as the cornerstone of health promotion and cholera prevention but this will require constant funding.

Prevention of cholera should include both strengthening the public water infrastructure and improved purification technologies at the household level [26]. While knowledge and enthusiasm for water treatment remains high, there should be an uninterrupted supply of water treatment products that are easily accessible and affordable, particularly for rural areas. As mentioned above, interventions should be accompanied by interpersonal communication with trusted sources to reinforce the value of clean water.

Although water treatment products are available in markets, household financial resources remain limited. In addition, rural populations in Haiti have grown accustomed to water treatment products being dispersed freely. It is not certain how easily a transition would occur to a market economy. Previous research has shown that an extended free trial period to use the product and perceive its benefits was important in the participants’ appreciation of what difference the product made in the lives of their family [13].

It is essential to build on the current knowledge base and recent positive experiences in using water treatment products. It will also take creative partnerships among the NGOs, private industry, and the Haitian government to create water and sanitation systems that are sustainable over time. These communities recognize what is needed in terms of potable water and sanitation but lack a sense of community determination to address these issues collectively. Building community enterprises to tackle these issues of poverty will take time and patience but should be initiated as quickly as possible.

## Supporting Information

S1 File Community Member FGD Interview Guide. (DOCX)

S2 File Community Health Workers FGD Interview Guide. (DOCX)
