# Supplementary material for: Perceptions of Health Communication, Water Treatment and Sanitation in Artibonite Department, Haiti, March-April 2012
Source: PLoS One. 2015 Nov 12;10(11):e0142778. doi: 10.1371/journal.pone.0142778 (PMC4642927; doi:10.1371/journal.pone.0142778)
Supplement: S1 File — (DOCX) [file pone.0142778.s001.docx]

**S1 File: Community Member FGD Interview Guide**

Hello, my name is _______________. I am with a group from the United States Centers for Disease Control and Prevention (CDC) and DINEPA and I will be leading the discussion today. We are public health workers trying to learn about water, sanitation, and hygiene practices in Artibonite to inform our future work. **[INTRODUCE THE TEAM MEMBERS]**

Before I explain what we will be doing, can you please introduce yourselves? **[Allow the participants to introduce themselves]**

We would like to ask you a few questions about the drinking water, hygiene, and sanitation practices in your community. We are asking these questions because we want to learn about these practices, the availability of safe water, and other household practices of people living in the Artibonite Department. The findings from this questionnaire may help improve access to water, sanitation, and hygiene services and products for the residents of this Department.

We will take the results of the FGDs back to DINEPA and your information will be used to help plan future programs to expand treatment of drinking water at both household and community levels. We are here only to talk with you and are not in a position to supply any water treatment products but we are eager to hear your thoughts. We hope that everyone can contribute to the discussion – there are no right or wrong ideas. Please respect everyone in the group and allow each other to have time to talk.

Before we go any further, I need to obtain permission from all of you for the discussion and also to record the discussion. We have note takers to write down your answers but we also want to record your talk so that we do not miss anything.

**[At this point, read the informed consent and obtain consent for the FGD and the recording of the FGD]**

Are there any questions before we start?

**_____________________________________________________________________________**

**I would like to start with some questions pertaining to health and communication.**

1. Do you know the name of your community health worker or brigadier? YES NO

1b) Do you have community meetings with the health work or brigadier? YES NO

1c) If YES, when was your last meeting and what was discussed?

1. Do you remember any meeting within the last six months discussing water treatment? YES NO

2a) What about sanitation? YES NO

2b) IF YES: when was this and what was discussed? [**PROBE**: were there any discussions about cholera and how to make your water safe to drink or talks about latrines– if so, please describe]

1. If I wanted to reach people in your community with a message, what would be the best way to reach them?

3a) What are the best ways to learn about health issues? [**PROBE**: can you tell me the best approaches to use for health messages? For example, by radio, through village leaders, by SMS on your cell phone?]

1. What are some of the health messages that you have heard?

4a) Did you understand the messages? YES NO

4b) Did any of the messages help you change a behavior? YES NO

4c) IF YES: please provide an example:

4d) Have you ever received a health message that you thought did not make sense to you or was not good for you or your community? YES NO

4e) IF YES: please provide an example

**Now I would like to ask you a few things pertaining to water treatment:**

1. How do people in this community tell whether water is safe for drinking?

1a) How did you learn about these methods?

1b) Of these various methods, is there one method that is better than all the other methods? YES NO

1c) IF YES: which one and please describe why you think it is the best method?

1d) What types of water do people in this area consider safe to drink?

1. Do people in this community routinely treat their water to make it safer? YES NO

2a) IF YES: what methods are used?

2b) Why do you think some people do not treat their water?

1. For the people who treat their water, where do they get the water treatment products?

3a) How do people learn about these products and where to get them?

3b) How available are these products? [**Probe:** is it easy to get these products?]

3c) Are there ever any times when the products are not available? YES NO

3d) If yes: please describe (for example, when are the products not available and do you know why they sometimes are available and why sometimes you cannot find them?)

1. For people who treat their water, what do they do when they run out of product? [Probe: where do you go to get additional water treatment products?]
2. How many of you buy water to drink from a private kiosk? (record number of people out of total number of people):

5a) For those of you who buy the water, is this your primary source of drinking water? [NOTE: ONLY ASK IF SOMEONE BUYS WATER FROM A KIOSK. SKIP Q IF NO ONE BUYS WATER]

5b) For those of you who buy water, why do you buy it? [NOTE: ONLY ASK IF SOMEONE BUYS WATER FROM A KIOSK. SKIP Q IF NO ONE BUYS WATER]

1. If people work outside of their home, for example, in the fields, where do they get their water to drink?
2. I am going to show you some water treatment products. **[SHOW SAMPLE PRODUCTS]**

Some are filters, some are powders and some are liquids. Please decide first which products are used in this area and then I would like everyone, as a group, to rank the products by their quality. Please put the product with the best quality on top. **[ALLOW TIME FOR THE GROUP TO RANK THE PRODUCTS – make a list of the products in ranked order]**

7a) Please describe why you ranked them in the order that you did (for example, what factors are important in quality?).

1. Are there any products that you would prefer that we have not shown to you? YES NO

8a) Please describe the product to me and tell me where you could get it:

8b) Why do you prefer this product over the other ones that I have shown you:

8c) Where did you learn about this specific product?

1. If you had to buy a water treatment product, which vendors or sources would you most trust?

9a) Why do you trust these vendors or sources?

**I would now like to talk about sanitation:**

1. Please describe anything new that you have learned about sanitation in the last year.
2. What are some of the things that causes diarrhea in children?

2a) What are some of the things that causes diarrhea in adolescents or adults?

1. Are latrines commonly found in this area at the household level (for example: is it more common for a house to have a latrine than to not have a latrine)? YES NO

3a) IF YES: please describe who owns the latrines (for example: are they owned by a single household, do households share latrines)?

3b) IF NO: why do you think that so few latrines are in this area (for example: is it due to lack of money, difficulties with building a latrine, lack of interest, lack of understanding of how using latrines is better for health)?

3c) What do people in your community say about those families that have a latrine?

3d) Are people who own a latrine considered to have more status or prestige in the community? YES NO

3e) IF YES: Why do you think so?

3f) How do people make decisions about having their own latrine versus sharing with another family? [**NOTE: ask only if households commonly share latrines]**

3g) Within a family, who makes a decision to have a latrine?

3h) How many people in this group have a latrine at their house? [**NOTE:**  count the number of people who raise their hands against total number in the group]

3i) What could be done to get more people to build their own latrines?

1. If someone does not have access to a latrine, where do they defecate and urinate?
2. **[START OF EXERCISE]** Here is a big sheet of paper. As a group, can you draw a map of your residential areas and indicate the main features of your community (for example, mark where most houses are located, mark where the churches are, the markets, any forested areas and any bodies of water, like streams). Please put an X where you have seen human feces located outside.
3. What are possible risks to health if people do not use latrines?
4. What are the top three priorities in this community for things that you would like to see improved?

7a) **[NOTE: ask only if sanitation is not on the list]**: I see that sanitation is not listed as a top priority. Please describe why you think other things are more important to your community than sanitation:
