# Supplementary material for: Perceptions of Health Communication, Water Treatment and Sanitation in Artibonite Department, Haiti, March-April 2012
Source: PLoS One. 2015 Nov 12;10(11):e0142778. doi: 10.1371/journal.pone.0142778 (PMC4642927; doi:10.1371/journal.pone.0142778)
Supplement: S2 File — (DOCX) [file pone.0142778.s002.docx]

**S2 File: Community Health Workers FGD Interview Guide**

Hello, my name is _______________. I am with a group from the United States Centers for Disease Control and Prevention (CDC) and DINEPA and I will be leading the discussion today. We are public health workers trying to learn about water, sanitation, and hygiene practices in Artibonite to inform our future work. **[INTRODUCE THE TEAM MEMBERS]**

Before I explain what we will be doing, can you please introduce yourselves? **[Allow the participants to introduce themselves]**

We would like to ask you a few questions about the drinking water, hygiene, and sanitation practices in your community. We are asking these questions because we want to learn about these practices, the availability of safe water, and other household practices of people living in the Artibonite Department. The findings from this questionnaire may help improve access to water, sanitation, and hygiene services and products for the residents of this Department. In addition to your group, we have been talking to groups of women and men in various communities.

We will take the results of the FGDs back to DINEPA and your information will be used to help plan future programs to expand treatment of drinking water at both household and community levels. We are here only to talk with you and are not in a position to supply any water treatment products but we are eager to hear your thoughts. We hope that everyone can contribute to the discussion – there are no right or wrong ideas. Please respect everyone in the group and allow each other to have time to talk.

Before we go any further, I need to obtain permission from all of you for the discussion and also to record the discussion. We have note takers to write down your answers but we also want to record your talk so that we do not miss anything.

**[At this point, read the informed consent and obtain consent for the FGD and the recording of the FGD]**

Are there any questions before we start?

**_____________________________________________________________________________**

**I would like to start with some questions pertaining to health and communication.**

1. Do you hold community meetings with the communities in which you work? YES NO

1a) IF YES: when was your last meeting?

1b) How often do you hold meetings?

1c) What did you discuss at the last meeting?

1. Did you hold any meeting specifically to discuss cholera? YES NO

2a) IF YES: what topics did the meeting include?

2b) **[ASK ONLY IF PREVENTION WAS NOT MENTIONED]:** did you include prevention of cholera in these meetings? YES NO

1. What have you found to be the most effective means of communicating health messages to your selected communities?
2. Can you please give us an example of what you thought was a ‘successful’ health campaign or a successful health education program (for example, “a health campaign for vaccines)?

4a) Please describe why you thought it was successful:

4b) Did anything change as a result of it and, if so, what changed?

1. Can you please give other examples of changes in behavior in your communities regarding a health issue?

5a) What do you think was the most important factor in changing the behaviors (for example: doing home visits, hearing the message multiple times, selecting the right person to deliver the message, etc.)?

**____________________________________________________________________________**

**I would now like to talk about treating water to make it safe to drink.**

1. How do people in your selected areas determine whether water is safe to drink?
2. Do people in your selected areas use the same water source for all activities of daily living (i.e., drinking, bathing, washing clothes or dishes)? YES NO

2a) What sources of water do they use?

2b) Why do you think that people use these sources?

1. For the people in your selected areas that treat their water, how do they do it?
2. Can you please describe the water treatment products that are in your selected areas, if any?

4a) From where do people in your area get these products?

4b) What do people do when they run out of these products?

4c) Please describe what you think is the best distribution system for water treatment products:

5) Do you think that there is a preferred system of treating water in your areas? YES NO

5a) IF YES: which one is preferred and why (NOTE: please identify which community area you represent when you answer)

5b) If people treat their water, when do people treat their water (for example: only when free products are distributed, only after receiving health messages, all the time, etc)?

1. What are the reasons for people not treating their water (for example: money, beliefs, distrust in the products, lack of availability, lack of access to a distribution site, etc.)?
2. If people in your area were to have to buy water treatment products, what would be the best way to encourage people to buy them?
3. If people work in the fields, from where would they get their drinking water while they are working?

**_____________________________________________________________________________**

**I would like to finish our discussion with some questions pertaining to sanitation:**

1. Have you conducted any health education session that has focused on sanitation in the last 3-6 months? YES NO

1a) IF YES: please describe it (for example: what topics were covered, how many people attended, who attended and where was it held)?

2) Do most people own latrines in your selected areas? YES NO

2a) If no: why not?

2b) In your area, what types of people own latrines?

2c) What do people say about those families that have a latrine (for example: only for the rich, owning latrines gives a family more prestige, latrines are not seen as a priority):

1. Are there latrines available at the clinics to which you refer your patients? YES NO

3a) IF NO: please describe why there are not available at the clinic:

1. Is sanitation perceived to be a problem in your selected communities? YES NO

4a) IF YES: please describe:

1. If people do not have access to a latrine, where do they go to urinate and defecate?
2. If you wanted to encourage latrine use, what would you tell people in your community?
